# Results of a phase II open-label, non-randomized trial of cisplatin chemotherapy in patients with *BRCA1*-positive metastatic breast cancer

**DOI:** 10.1186/bcr3231

**Published:** 2012-07-20

**Authors:** Tomasz Byrski, Rebecca Dent, Pawel Blecharz, Malgorzata Foszczynska-Kloda, Jacek Gronwald, Tomasz Huzarski, Cezary Cybulski, Elzbieta Marczyk, Robert Chrzan, Andrea Eisen, Jan Lubinski, Steven A Narod

**Affiliations:** 1 Department of Genetics and Pathology, International Hereditary Cancer Center and Clinic of Oncology Pomeranian Medical University, ul. Rybacka 1, Szczecin, 70-204, Poland; 2 Sunnybrook Odette Cancer Centre, University of Toronto, 2075 Bayview Avenue. Toronto, ON M4N 3M5 Canada; 3 Department of Medical Oncology, Marie Sklodowska Clinic, Curie Memorial Institute, Garncarska 11, Krakow, 31-115, Poland; 4 Department of Oncology, Regional Oncology Hospital, Strzalowska 22, Szczecin, 71-730 Poland; 5 Department of Genetics and Pathology, International Hereditary Cancer Center, Pomeranian Medical University, ul. Rybacka 1, Szczecin, 70-204, Poland; 6 Department of Radiology, Jagiellonian University Collegium Medicum, Kopernika 19, Krakow, 31-501, Poland; 7 Women's College Research Institute, University of Toronto, 790 Bay Street, Toronto, ON M5G 1N8, Canada

## Abstract

**Introduction:**

The purpose of this investigation was to evaluate the efficacy of cisplatin chemotherapy in *BRCA1 *mutation carriers with metastatic breast cancer.

**Methods:**

In a phase II, open-label study, 20 patients with metastatic breast cancer who carried a mutation in *BRCA1 *were treated with cisplatin 75 mg/m^2 ^intravenously every 3 weeks as part of a 21-day cycle for 6 cycles. Restaging studies to assess response were performed after cycles 2 and 6, and every three months thereafter.

**Results:**

Between July 2007 and January 2009, 20 patients were enrolled. Baseline characteristics were as follows: 65% had prior adjuvant chemotherapy, 55% had prior chemotherapy for metastatic breast cancer; mean age was 48 years (ranges 32 to 70); 30% estrogen receptor (ER) or progesterone receptor (PR)+, 70% ER/PR/Human Epidermal Growth Factor Receptor 2 (HER2)- and 0% HER2+. Overall response rate was 80%; nine patients experienced a complete clinical response (45%) and seven experienced a partial response (35%). Overall survival was 80% at one year, 60% at two years and 25% at three years. Four of the 20 patients are alive four years after initiating treatment. The median time to progression was 12 months. The median survival from the start of cisplatinum treatment was 30 months. Cisplatin-related adverse events, including nausea (50%), anemia (5%) and neutropenia (35%) were mostly mild to moderate in severity.

**Conclusions:**

This phase II study demonstrates that cisplatin chemotherapy has high activity in women with a *BRCA1 *mutation and metastatic breast cancer and is generally well tolerated.

**Trial registration:**

This trial is registered retrospectively on the clinical trials website ClinicalTrials.gov. The identifier is NCT01611727.

## Introduction

Among women with a *BRCA1 *mutation and breast cancer, choice of chemotherapy is a critical issue. There are emerging data which suggest that mutation carriers may respond differently than non-carriers to particular agents [1-3]. *BRCA1*-associated cancers differ from non-hereditary cancers for a range of pathologic and molecular factors, including tumor grade and histologic appearance [4-7]. Several studies have shown that the response to treatment for women with a *BRCA1*-associated breast cancer reflects the underlying tumor biology, in particular, the impairment of the DNA damage response and repair pathways, and that it is possible to exploit the sensitivity of *BRCA1*-associated cancers to DNA damage [8,9].

This study was performed as a component of a large, multi-center research program conducted in Poland at the Pomeranian Medical University, which is designed to characterize the hereditary burden of breast cancer in the country and to identify and evaluate means of prevention, screening and treatment for women with *BRCA1 *mutations. There are three *BRCA1 *founder mutations in Poland (5382insC, C61G and 4153delA), which account for the great majority of all *BRCA1 *mutations in Polish families [10-12].

We have previously reported, in a small study of *BRCA1*-positive women with early breast cancer, that a high rate of complete pathologic response was achieved using cisplatin chemotherapy as a single agent in the neo-adjuvant setting [13]. It is equally important that we evaluate the benefit of cisplatin in women with disseminated breast cancer, including those who have previously been treated with one or more chemotherapy regimens. This study was undertaken to evaluate the efficacy of cisplatin chemotherapy in *BRCA1 *carriers with metastatic breast cancer. The primary objective was to determine the objective response rate of cisplatin in *BRCA1 *carriers with metastatic breast cancer. The secondary objectives were to determine three-year survival and to evaluate the toxicities of cisplatin in *BRCA1 *carriers with metastatic breast cancer.

## Materials and methods

### Patient eligibility

Female patients age ≥18 years, with measurable (defined by Response Evaluation Criteria in Solid Tumors (RECIST) criteria [14]) metastatic (stage IV) breast cancer, and who were known to carry a *BRCA1 *mutation, were eligible. In addition, the following were required: adequate hematologic, renal and hepatic function; adequate recovery from recent surgery and/or radiation therapy; recovery from all prior treatment-related toxicities (to grade <2 according to National Cancer Institute Common Toxicity Criteria, Version 3.0, except alopecia); life expectancy of at least 12 weeks; and an Eastern Cooperative Oncology Group (ECOG) performance status of 0 or 1. Patients could have received up to four prior chemotherapies for metastatic disease. Patients with known brain metastases were not eligible. Patients previously treated with a platinum-based chemotherapy were not eligible.

The protocol was approved by the Ethics Committee of the Pomeranian Medical University with jurisdiction over the specific sites that registered patients on the study. The study was approved by Ethics Committee of the Pomeranian Medical University BN-001/83/07

Patients were recruited from three academic hospitals in Poland. All patients provided written informed consent before enrollment.

### Study design and treatment

This was a Phase II, open-label, non-randomized, multi-center study. Cisplatin (Ebewe, Austria) chemotherapy was administered as a 75 mg/m^2 ^intravenous (IV) infusion every three weeks, for six cycles. Dexamethasone (8 mg) (PABI-Dexamethason, Polfa, Pabianice, Poland) was administered once daily for three days after chemotherapy. Ondansetron (Zofran™) (Zofran, GlaxoSmithKline, Great Britain was used for anti-nausea prophylaxis.

A total of 18 of the 20 patients received additional lines of chemotherapy subsequent to platinum therapy at the time of progression. These included AT (Doxorubicin, Ebewe, Austria and Docetaxel, Sanofi-Aventis, Poland) (eight patients) ACT (Doxorubicin, Ebewe, Austria and Cyclophosphamide, Baxter, Poland and Docetaxel, Sanofi-Aventis, Poland) (two patients), FAC (5-Fluorouracil, Ebewe, Austria and Doxorubicin, Ebewe, Austria and Cyclophosphamide, Baxter, Poland) (two patients) and AC (Doxorubicin, Ebewe, Austria and Cyclophosphamide, Baxter, Poland) (one patient. Four patients received an aromatase inhibitor after platinum therapy.

### Assessments

Baseline assessments included the following: medical history, complete physical examination, assessment of performance status on the ECOG scale, physical and radiological examination of the tumor, and complete blood count (CBC) and complete metabolic profile. Hormone (estrogen receptor (ER)/progesterone receptor (PR)) and Human Epidermal Growth Factor Receptor 2 (HER2) receptor status were performed locally. During treatment, these assessments were performed in the same manner at the start (Day 1) of each cycle, except radiological examination of the tumor which was performed after Cycle 2 and Cycle 6, and every three months thereafter.

### Response criteria and toxicity

Objective tumor response rate, defined as the percentage of patients who achieved a complete response or partial response by RECIST criteria [14], was the primary efficacy end-point. Radiological assessments were carried out via computerized tomography at baseline, after two cycles (6 weeks), after six cycles (18 weeks) and every 3 months thereafter. Secondary end points included one-year survival and toxicity. Safety assessments included adverse events, clinical laboratory tests, ECOG patient safety and physical examinations and vital signs. Adverse events were graded according to the National Cancer Institute Common Toxicity Criteria (CTC), version 2.0. After completion of six cycles of cisplatin, the patient was seen in the clinic every two weeks for eight weeks and every four weeks thereafter. A repeat CT scan was done at three-monthly intervals to evaluate progressive disease.

### Statistical analysis

The primary objective of this study was to determine the overall response rate of cisplatin in metastatic breast cancer patients with a known *BRCA1 *mutation (complete or partial response). Secondary objectives for this study included estimating the one-, two- and three-year rates of overall survival and the evaluation of toxicity. The intent-to-treat (ITT) population was defined as all eligible patients enrolled in the study that had no major violations of protocol inclusion and/or exclusion criteria.

The response rate was calculated as the number of responders (best study response recorded as complete response or partial response) divided by the number of patients enrolled. Survival was calculated using the Kaplan-Meier methods. Patients were followed from the date of first receiving cis-platinum until the date of first evidence of progression, or the date of death, depending on the analysis.

## Results

### Patient characteristics

Between July 2007 and January 2009, 20 women were enrolled in the study. No prospective patient was found to be ineligible or declined to participate. All study patients had been tested previously for the presence of three *BRCA1 *founder mutations and had been found to be positive. Eighteen patients were treated in Szczecin and two were treated in Krakow.

Patient characteristics are summarized in Table 1. This patient population was notable for its young age (median age 48 years), predominance of 5382insC *BRCA1 *mutations, and predominance of triple-negative cancers (70%). More than one-half of the patients had been treated previously with chemotherapy for metastatic disease. The median disease-free interval from the first diagnosis of breast cancer until the time of metastatic disease was 2.2 years. No woman had a prior history of ovarian cancer or of another form of cancer.

**Table 1:** Characteristics of patients in the study

**Age, years**	
Mean (range)	48.0 (32 to 70)
**Type of BRCA1 mutation **	
5382insC	13
C61G	6
153delA	1
**Year of initial treatment**	
2000 to 2003	2
2004 to 2006	7
2007 to 2009	11
**Estrogen receptor**	
Positive	5
Negative	15
**Progesterone receptor**	
Positive	3
Negative	17
**HER2 status**	
Positive	0
Negative	20
**Histology**	
Ductal	11
Medullary	7
Other	1
Missing	1
**Prior adjuvant chemotherapy**	
Yes	13
No	7
**Sites of metastases**	
Liver	10
Lung	9
Bone	3
Other	11
**Number of sites of metastases**	
1	12
2	4
3	2
4+	2
**Prior chemotherapy for metastatic disease**	
Yes	11
No	9
**Line of therapy of metastatic disease**	
1	9
2	2
3	8
4	1

Of the 20 patients enrolled in the study, 17 completed six cycles of cisplatin chemotherapy. Two patients did not complete treatment due to disease progression. One patient discontinued therapy after four cycles due to grade 4 neutropenia. Five patients delayed treatment due to neutropenia or anemia. One patient required blood transfusion. One patient continued to exhibit tumor shrinkage after six cycles and thus, at the treating physician's discretion, received nine cycles of chemotherapy.

### Treatment outcomes

**Figure 1:**
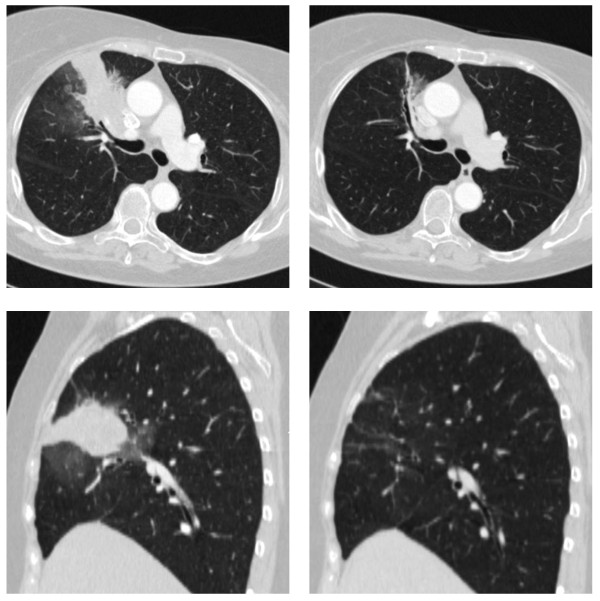
**CT images of pre- and post-treatment for patient experiencing a complete response**. Upper left: pre-treatment axial-mediastinal window. Upper right: post-treatment axial mediastinal window. Lower left: pre-treatment sagittal-mediastinal window. Lower right: post-treatment sagittal-mediastinal window.

Responses of the patients are summarized in Table 2. The overall response rate was 80% (complete response 45%; partial response 35%). Those patients who responded included 8 of 9 women for whom cisplatin was their first-line treatment for metastatic disease (89%) and 8 of 11 women (73%) who had previously been treated for metastatic disease. A partial or complete response was achieved in 11 of 12 patients with disease at one site (92%), and in 5 of 8 patients with disease at multiple sites (63%). A partial or complete response was achieved in 12 of 15 ER-negative patients (80%), and in 4 of 5 ER-positive patients (80%). A complete response was achieved in 8 of 15 ER-negative patients (53%), compared to only 1 of 5 ER-positive patients (20%). A complete response was achieved in 7 of 14 triple-negative breast cancer patients (50%). A complete response was observed in 5 of 12 patients with a 5382insC mutation and in 4 of 7 patients with a C61G mutation (OR = 0.54; *P *= 0.6; Fishers exact test). Of the nine women who had a complete response, all were determined on the basis of the scan four months after stopping treatment. An example of CT scans, pre- and post-treatment for a woman who experienced a complete response is shown in Figure 1.

**Table 2:** Distribution of response types among patients treated with cis-platinum

Response	Number	Evidence of progression	Time to progression Median (months)	Number deceased
Complete	9	9	17	6
Partial	7	7	8	6
Stable	3	3	3	3
Progressive	1	1	1	1

### Progression-free survival

**Figure 2:**
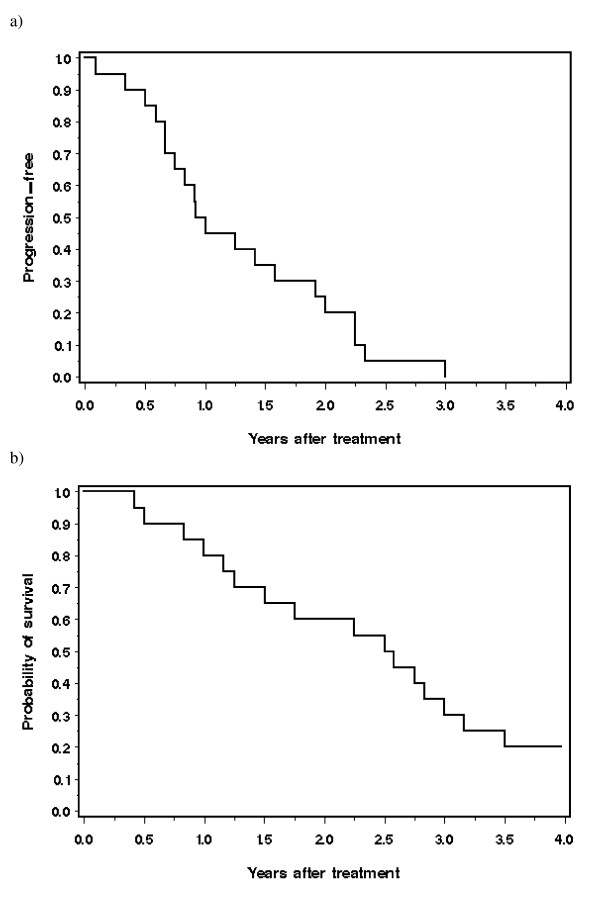
**Progression after initiation of cisplatin treatment**. **b) **Survival after initiation of cisplatin treatment.

All 20 patients have now experienced progression. The median time to progression was 12 months (range 1 to 36 months). The proportion of patients experiencing progression was 55% at one year, 80% at two years and 95% at three years (Figure 2a). Among the nine patients who experienced a complete response, the median time to progression was 17 months.

### Overall survival

The actuarial survival rate for the cohort was 80% at one year, 60% at two years and 25% at three years (Figure 2b). The median survival was 30 months. Four of the 20 patients are currently alive (20%). Each of these four patients has survived four years or more from the initiation of cisplatinum treatment. Of these, three experienced a complete response and one a partial response. The times elapsed from initiation of treatment for each of the four living patients was 50, 50, 54 and 62 months. Four patients had a time to progression of two years or longer. They are described in Table 3.

**Table 3:** Characteristics for four study subjects that had a time to progression of two years or longer

	Case number			
	1	13	6	2
**BRCA1 mutation**	C61G	C61G	5382insC	5382insC
**Age at treatment**	56	34	48	66
**Estrogen receptor**	Negative	Negative	Positive	Negative
**Progesterone receptor **	Positive	Negative	Positive	Negative
**HER2 status**	Negative	Negative	Negative	Negative
**Site of metastases**	Lung	Lung, liver	Liver	Liver
**Number of sites of metastases**	1	2	1	1
**Prior adjuvant chemotherapy **	Yes	Yes	Yes	Yes
**Line of therapy of metastatic disease**	1	3	1	2
**Response**	Complete	Complete	Partial	Partial
**Time from treatment to progression (months)**	36	27	27	28

### Toxicity

Treatment-related toxicities are summarized in Table 4. Cisplatin was generally well tolerated. Consistent with previous cisplatin studies, the most common adverse events were nausea (50%), anemia (5%) and neutropenia (35%). A grade 3/4 adverse event occurred in two patients; for both of these, cisplatin was the third-line treatment. A total of 20% of the patients required dose adjustment and/or treatment interruption because of anemia or neutropenia. One patient discontinued cisplatin due to neutropenia.

**Table 4:** Toxicities experienced by the patients

Toxicity	N (%)
Neutropenia	
	
Grade 1	1
Grade 2	4
Grade 3	1
Grade 4	1
	
Any	7 (35%)
	
Nausea/vomiting	
	
Grade 1	4
Grade 2	6
	
Any	10 (50%)
	
Anemia	
	
Grade 1	0
Grade 2	0
Grade 3	1
	
Any	1 (5%)
	
Neurotoxicity	
	
Grade 1	2
Grade 2	4
Grade 3	0
Grade 4	0
	
Any	6 (30%)

## Discussion

There has been a recent resurgence in interest in evaluating platinum-based chemotherapies in patients who are known to have an inherited deficiency in DNA repair [15]. An early study evaluating the role of cisplatin in the first-line therapy of metastatic breast cancer showed an overall response rate of 47% [16]. In our study, a partial or complete clinical response was achieved in 16 of 20 (80%) patients with a *BRCA1 *mutation and metastatic breast cancer who received cis-platinum chemotherapy as a single agent. The median survival time from the start of cisplatinum treatment was 30 months.

There are few studies which report on the survival experiences of cohorts of *BRCA1 *patients. In a recent study from the Netherlands, Kriege *et al*. reported on 93 women with a *BRCA1 *mutation and metastatic breast cancer who were treated with conventional chemotherapy (CMF (Cyclophosphamide, methotrexate, 5-Fluorouracil, or anthracycline-based) [17]. The objective response rate was 66% and the median progression-free survival was 7.6 months. In our study, the median time to progression was 12 months. The median overall survival in the Kriege study was 15 months, versus 30 months in our study. In an early report of metastatic breast cancer patients treated with the PARP inhibitor olaparib, 33 *BRCA1 *carriers were included [18]. The median time to progression was approximately four months for those treated with 100 mg of olaparib twice a day and was seven months for those treated with 400 mg twice a day (specific figures for the *BRCA1*-positive subgroup are not given). Thus, the patients in the current study who have been treated with cisplatin appear to have superior outcomes to these historical controls. However, it is difficult to compare the survival experience of different patient cohorts who may have different disease patterns and different baseline characteristics. The numbers of *BRCA1 *carriers in these studies are not large and some of the variation may be due to random fluctuation.

It is of interest to try to identify predictors of response in these patients to determine which women are more likely to benefit from cisplatin chemotherapy. Our study was too small to be definitive in this regard, but it is of interest that the past use of chemotherapy for metastases was associated with a lower response rate, as was the presence of disease at multiple sites. Also, only 1 of 5 patients with ER-positive breast cancer had a complete response, compared to 8 of 15 ER-negative breast cancers. However, the numbers of patients in these subgroups were small and it is too early to make definite conclusions in this regard.

The results of this study are consistent with our earlier results of the use of cisplatin as a neo-adjuvant treatment of breast cancer. In a study of 102 *BRCA1 *carriers who received neo-adjuvant chemotherapy, 10 of 12 women with a *BRCA1 *mutation who were given cisplatin experienced a complete pathologic response (83%), compared to 14 of 90 patients (16%) treated with other regimens (*P *< 0.001; exact test) [19]. To date, we do not have experience in the use of cisplatin for patients with metastatic breast cancer who had earlier received cisplatin in the adjuvant or neo-adjuvant setting. Studies of ovarian cancer patients might be more informative in this regard. Tan *et al*. found that patients with ovarian cancer and a *BRCA1 *mutation often responded to cisplatin on multiple occasions during the course of their treatment [20]. This is in keeping with an earlier study by Cass *et al*., who reported that women with ovarian cancer and a *BRCA1 *mutation were more likely than non-carriers to respond well to cisplatin [21]. Swisher *at al*. documented revertant mutations in four of the six recurrent platinum-resistant ovarian cancers [22]. These secondary mutations restored the reading frame of the *BRCA1 *protein. We do not have data on the mechanisms of eventual resistance to cis-platinum and it will be of interest to establish whether revertant mutations are also a source of platinum resistance in breast cancer.

In a mouse model which expressed *BRCA1 *mutations in mammary tissue, Drost *et al*. found that tumors associated with the C61G mutation had, on average, a poorer response to platinum-based drugs than did mice with tumors that were homozygous null for *BRCA1 *[23]. There is no evidence in our clinical study that patients with the C61G mutation were less responsive to platinum than were patients with the more common 5382insC mutation.

There are several limitations to this study. This is a relatively small sample of patients (*n *= 20) and they originate from three institutions. Nevertheless, this is among the largest series of *BRCA1 *carriers with metastatic breast cancer to be studied to date, and the underlying Polish population is relatively homogeneous. We conducted computed tomography (CT) scans after two and six cycles due to the constraints of the institutions and, therefore, it is challenging to accurately assess stable disease, duration of response and time to progression. Ideally, we would have had more frequent scans and we would have assessed disease at 16 weeks as well. However, the data regarding overall survival are perhaps the most striking and this endpoint is straightforward to evaluate. A total of 18 of the 20 patients were treated with subsequent lines of chemotherapy after platinum therapy and, therefore, it is not possible to dissociate the effect of the cis-platinum therapy from that of other drugs.

## Conclusions

In conclusion, our data suggest that chemotherapy regimens with cisplatin may benefit patients with metastatic breast cancer and a *BRCA1 *mutation. These early results are encouraging, but they should be confirmed in a larger randomized controlled trial.

Future studies should evaluate the potential of using this therapy for the treatment of hereditary breast cancer due to other genes, including *BRCA2*. Given that there do not appear to be founder mutations in *BRCA2 *in Poland, it will be necessary that these studies be conducted in other countries (or provinces) where *BRCA2 *founder mutations are prevalent (for example, Quebec) [24]. Other agents that target DNA repair deficiency may also prove to be beneficial in this subgroup of patients.

## Abbreviations

ACT: adriamiycin/cyclophosphamide taxol; AT: adriamycin/taxol; CBC: complete blood profile; CTC: Common Toxicity Criteria; ECOG: Eastern Cooperative Oncology Group; FAC 5: flurouracil, adriamycin, cyclophosphamide; HER2: Human Epidermal Growth Factor Receptor 2; ITT: intent-to-treat; OR: odds ratio; pCR: pathologic complete response; RECIST: Response Evaluation Criteria In Solid Tumors

## Competing interests

The authors declare that they have no competing interests.

## Authors' contributions

TB supervised clinical activities and data collection, and drafted and edited the manuscript. RD helped with study design and edited the manuscript. PB, MF-K and EM recruited patients and helped with clinical monitoring. JG, TH and CC performed genetic testing and recruited patients. RC acted as a clinical advisor on medical imaging, and interpreted and provided images. AE reviewed the manuscript and acted as the clinical advisor for medical oncology. JL was the principal investigator of all activities in Poland. SN was the lead investigator that designed the study, performed the analysis and edited the manuscript. All authors read and approved the final manuscript for publication.
